# Valid knowledge of performance provided by a motion capturing system in shot put

**DOI:** 10.3389/fspor.2024.1482701

**Published:** 2025-01-17

**Authors:** Stefan Künzell, Anna Knoblich, Annika Stippler

**Affiliations:** Institute of Sports Science, University of Augsburg, Augsburg, Germany

**Keywords:** feedback, experimental studies, knowledge of performance, validity, objectivity

## Abstract

Extended feedback on knowledge of performance in sports techniques is very challenging and requires a high level of expertise. This poses a significant problem for experiments on providing extended feedback, as it is essential to ensure that the “correct” feedback is given for it to be effective. In this study, we investigate whether the correct feedback can be determined based on kinematic data. Ten participants and one model were recorded during shot put using a Motion Capturing (MoCap) system and simultaneously captured on video. The videos were analysed by two experts, and the two most critical errors were noted. By qualitatively comparing the deviations of the participants from the model, the experts’ error feedback was identified in the motion curves of the MoCap system. The expert feedback for two participants was sealed in an envelope. In a qualitative analysis of the motion data, the error feedback was then determined and subsequently compared with the experts’ feedback. These error feedbacks largely matched. It was shown that, in principle, it is possible to extract errors from the kinematic angle and distance curves of the movement. This study opens the door to an automated version of the qualitative assessment of movements by AI. Further research can now focus on the topic of conveying AI-generated feedback. This could then also provide a valid foundation for experiments on the effects of knowledge of performance.

## Introduction

A wide variety of professions, some of them very well paid, are engaged in helping their fellow human beings to move in a purposeful way: physical education teachers, physiotherapists, music masters, coaches, to name a few. One tool often used in this process is instruction and feedback. While instructions are typically given before movement initiation, feedback refers to a movement that has already been performed. Nevertheless, feedback usually includes, at least implicitly, instructions about what to do if the movement is executed again. The feedback: “The ball was thrown 10 cm too short” refers to the result of an executed movement but is closely related to the instruction “Throw the ball 10 cm further in the next attempt”. In the following we will talk about feedback, but many of the considerations are easily transferable to giving instructions.

Motor learning, i.e., the acquisition of the solution to a movement problem, is certainly possible through trial and error. But sometimes feedback from the teacher can shorten the learning process siginifcantly. However, giving the appropriate feedback is not a simple matter—it may require a high level of expertise (and is also the reason for the sometimes extraordinarily high salaries of feedback-givers). Human generated feedback may suffer from a possible lack of expertise, subjective perception and weighting of various movement errors, limitations in the spatial and temporal resolution of human perception and the consideration of extraneous factors. In this paper, we argue that especially the required expertise is the reason why only very simplified movements have been studied in feedback research, which considerably limits the validity of the research results.

In feedback research, a distinction is made between external (or augmented) and task-intrinsic feedback on the one hand, and between feedback of the result and feedback on the performance on the other hand, also known as knowledge of result (KR) and knowledge of performance (KP), respectively. While KR can be measured objectively but often includes trivial information, KP is often subjective and dependend on the feedback givers expertise but includes helpful information for learning sport techniques. For these reasons, in research mainly KR is studied, while in practice mainly KP is relevant (1, 2).

### Task-intrinsic and external feedback

Task-intrinsic feedback is “the sensory feedback that is naturally available while performing a skill” (1, p. 333), that is all feedback that is perceived during the execution of a movement and possible delayed movement effects by the body's own sensors without transformation by third parties. This type of feedback is always available to the athletes. Kinaesthetic sensors provide information about muscle tensions and joint positions, haptic sensors about the nature of objects touched as well as the force effect they exert. Auditory and visual sensors, on the other hand, provide information about changes in the environment caused by one's own movement—but also about changes that were not caused by one's own movement.

Task-intrinsic feedback has at least two functions. On the one hand, it can inform during the movement whether the solution of the movement task is progressing. For example, if you want to drink out of a plastic cup, during your grasping movement, visual perception can feedback the difference between the grasping hand and the cup. Haptic feedback then provides information about the gripping strength, whether the strength with which we grasp the cup is sufficient to keep it from slipping through our fingers, but also not too great for the plastic cup to deform and break. This feedback-based control is called closed-loop control. On the other hand, intrinsic feedback can be used to determine the success or failure of a movement. For this purpose, the perceived sensory consequences are matched with the intended and the anticipated sensory consequences (3). According to the ideomotor principle (4, 5), it is assumed that the intended goals of a movement are encoded as imagined sensory consequences, which then allows comparison with the consequences perceived at the end of the movement. Furthermore, in a forward model, the sensory consequences of the emitted efferents are modelled (6). They are used to plan movements and, if necessary, to attribute an error in the movement outcome to the error in the movement production (7).

External feedback is given by third parties, usually verbally by a trainer and/or visually by video recordings. Because it is added to the always available task-intrinsic feedback, it is often called augmented feedback. We prefer the term external feedback, because the external source is the defining criterion, not the augmentation (A wrong or useless external feedback might not augment, but diminish task-intrinsic feedback, but is external nevertheless). What influence does external feedback have on the learning process? On the one hand, feedback can influence the intended sensory consequences. An admittedly very simple example is the reference to certain rules in sports. For example, an athlete in the high jump could arrive at a movement solution with a two-legged jump by trial and error. The feedback “you may only jump with one leg” leads to other intended sensory consequences in the movement process. However, feedback on recognized good movement solutions is also useful. For example, the feedback in basketball “try to give the ball a backward rotation during the free throw” will considerably shorten a learning process by trial and error.

Secondly, feedback can also be aimed at the perception of the initial situation. In sport climbing, for example, it is not uncommon for beginners to overlook certain holds under the psychological strain (8). The feedback to use that certain hold can be necessary for success.

Third, feedback can also refer to the sensory consequences that have occurred. When beginners in floor gymnastics produce a scale forward, it is not uncommon for the rear leg to not be fully extended. However, inexperienced gymnasts might not notice this because they interpret the associated internal feedback as a stretched leg. Extrinsic feedback e.g., by a video recording can easily remedy this.

With external feedback, it is the athletes’ task to find the appropriate changes in efferences. This is only successful with an already reasonably well-developed forward model. Regarding the feedback “10 cm too short”, it is quite easy to set the efferences for a slightly wider throw. Therefore, the feedback of the result is also helpful here. However, the feedback “You fell” presented to a skier after a fall is not very helpful, although it is of course still correct. Here a KR is not sufficient, instead KP must be given. It would make sense here to give feedback that addresses the cause of the fall. However, as there are many different reasons for falling while skiing, the feedback requires the great expertise of the coach.

Sports science studies on feedback now mostly refer to feedback of the movement outcome, where it is relatively clear through a well-developed forward model which changes in efferences are needed for a better movement outcome. This relates to studies of accuracy of feedback, frequency of feedback, self-selected feedback, and the like. These studies are correct and important, but they probably do not allow us to draw conclusions about the laws of feedback, where athletes do not have a good forward model and therefore cannot transform feedback into a change in efferences. They simply have no idea what to do. Thus, it would be necessary to also investigate knowledge of performance, which is most of the feedback in practice. However, in the case of meaningful feedback from KP, the expertise problem arises. Hence, in experiments with external KP, results are probably confounded by the quality of the feedback, because it is hard to determine if the individually best external feedback has been given.

We are not the first to draw attention to this problem. Schmidt and Young already complained in 1991 that feedback research up to that time was primarily concerned with KR and that this was often simply equivalent to the movement result (9). Thus, experimental participants often had to perform precision tasks, but where the target was not visible. They received the deviation as “extended” feedback from the experimenter. In addition, Schmidt and Young complained that no information was given about what to do next and that the tasks were very simple (9). They suggested to use instead a new standard task, a coincident timing task with a specific device they had designed. However, they were not successful in establishing their tasks as a standard experimental design. At about the same time in Germany, a group of researchers around Daugs (10, 11) proclaimed a more complex standard task, the whole-body wave from rhythmic gymnastics, with which they investigated various regularities in the implementation of KP. Using joint angle trajectories from eleven joints, they measured the deviation of the measured motion from a model. One problem was that the analysis of the data was very time-consuming, so it was not available shortly after the movement. Thus, while they were able to objectively demonstrate improvements from pretest to pos*t*-test, their feedback was solely related to the presentation of videos taken during the execution of the movement. Objective verbal feedback resulting from the data was not available to them.

Since the 90s of the last century, however, a lot has happened in the field of automated motion analysis (motion capturing, MoCap). Through various technological advancements, motion capturing data is now available in real time. This opens the possibility that even complex movements can be examined. With the help of MoCap, objective data with a high spatial and temporal resolution can bei provided. This opens the door for an analysis by Artificial Intelligence and allows the use of complex sporting techniques to analyze different feedback methods, such as the frequency or timing of feedback.

### Aim of the study

Our study aims to demonstrate that people, using their natural pattern recognition abilities, can qualitatively identify differences between the kinematic curves of the participants and the model, which reflect the qualitative feedback of experts. Moreover, they are able to identify previously unknown error pattern in the overall behavior of the athletes by qualitatively analysing kinematic curves delivered by a MoCap system.

Once we have shown this, the next step is to feed the MoCap data into artificial neural networks so that, through deep learning, an artificial intelligence can detect movement errors based on differences in the curves and provide feedback.

The goal is to show that it is, first, fundamentally possible to identify expert qualitative feedback in kinematic data, and second, that it is also fundamentally possible to generate qualitative feedback from the kinematic curves.

## Method

The pilot investigation presented below is a feasibility study. We investigated by a qualitative analysis whether the error characteristics of a movement named by expert raters can also be found in the objective data provided by the MoCap. In a second step, we analyse—again qualitatively—if it is possible to use the MoCap data to infer the main errors in the movement performed and thus find the objectively best KP.

We choose the glide technique in shot put. The participants wore a special suit equipped with 13 acceleration sensors (Xsens Awinda), which provides the kinematic data of all body parts and joint angles with the help of software produced for this purpose (Xsens Analysis).

### Participants

A total of 10 participants (five male, five female; volunteer sampling), one male model and two expert raters (one male, one female) participated voluntarily in the study. The 10 participants were pre-service physical education teachers from the University of Augsburg between their 2nd and the 4th year of education (age M = 23.0 years, SD = 1,61 years). They had completed a course in athletics lasting at least one semester, where they learned the basic form of the shot put technique to such an extent that they have reached the level of freeing the degrees of freedom (12). However, none of the participants trained regularly in shot put technique or practiced athletics at a competitive level. Due to better comparability, only right-handed athletes participated in the study. All participants and the model thus put shot with their right hand.

The model has 10 years of athletic decathlon experience at a state level. His execution of the shot put is a technically high-quality movement. In the course of the work, his execution (target value) is compared with the respective execution of the participants. For this purpose, in every movement of each participant and the main model for key time points were identified, the start of the glide phase, the end of the glide phase, the power position and the actual shot. These time points were determined using the avatar and the three-dimensional view of the program. The following criteria were considered. The start of the glide phase began as soon as the Center of Mass (COM) moved solely in the direction of the shot. The end of the glide phase was marked by the ground contact of the right leg. The throwing stance was reached when the participant was positioned directly before the throw. The throw itself was defined by the full extension of the arm and the flicking of the wrist. To make the kinematic curves comparable, the model's curve was compressed or stretched so that the four time points for the model and each respective participant were reached at the same times.

The expert raters are both experienced university teachers and active in teaching and exam preparation in the field of athletics, and have extensive expertise in the technique of shot put and its didactics (male expert 5 years experience, female expert 26 years experience). All participants, the model and the expert raters gave their written informed consent to participate in the study. The data protection guidelines of the University of Augsburg and the Declaration of Helsinki were observed.

### Procedure

After the participants and the model had warmed up and repeated the shot put glide technique, they were fitted with the Xsens MVN Link motion suit (Movella) and all motion trackers were attached to the corresponding body parts. After the calibration procedure, the participants performed two shot puts. These were recorded by the MVN Analyse motion capturing (MoCap) system (Xsens, Movella) and filmed from the side with a video camera. Furthermore, we measured the distance of the respective shot put.

In this way, we recorded a total of 20 trials both by the Xsens program and on video. We selected 15 shot puts from the available trials, for which both recordings worked technically flawlessly. The videos of the 15 shot puts recorded by the camera were shown to two independent expert raters. They were able to watch the video repeatedly, pausing at crucial times and playing the video at slowed speed. We informed them about the distances of the shots for each trial. We asked them to write down the main error and the second most important error of 13 videos, they were handed to the experimenter. They analysed the remaining two videos without the experimenter's presence. They collected these answers in an envelope and sealed it. Please note that these are qualitative error descriptions that cannot be statistically analyzed. For example, the main error written by Expert 1 for Participant 10 was: “Pelvis is not upright when reaching the power position” and the second error “tilting of the shoulder axis”, whereas Expert 2 wrote “No proper power position” and “No blocking of the left side, tilting of the upper body”. We condensed these statements to “Tilting of the shoulder axis in the push-off” (Table 1), but do not want to judge if error statements of the two raters actually match exactly or not.

**Table 1:** Matching variables for motion analysis to the respective error pattern.

Error pattern	Variables for motion analysis	Phase of interest
Tilting of the shoulder axis in the push-off	Position of the right and left shoulder (z-coordinate corresponds to the height)	Reaching the push-off position
Low extension of the left leg during the gliding movement	Flexion/extension of the left knee joint	Gliding movement
Right foot is not under the COM at the end of the glide movement	Position of the toes of the right foot and position of the body's centre of gravity (x and y coordinates).	
Open upper body during the gliding movement	Axial rotation of the T1-C7 articulated joint and the C1 head articulated joint	Gliding movement
Lowering of the upper body and the right hand when reaching the power position	Position of the sternum and the right hand (z-coordinate corresponds to the height)	Reaching power position
Elbow is lower or behind the hand during push-off	Position of the right hand and forearm (x and y coordinates)	Reaching push-off
Shot putting without the help of leg power	Flexion/extension of the right knee joint	Reaching push-off
Too little distance between the left and right foot when reaching the power position	Position of the toes of the right and left feet (x- and y-coordinate)	Reaching power position
Right leg is not turned in when reaching the power position	Internal/external rotation of the right hip joint	Reaching power position
Pelvis is not upright when reaching the power position	Flexion/extension of the vertical pelvis (vertical pelvic position)	Reaching power position
Extending movement of the left leg upwards during the gliding movement	Position of the left foot (z-coordinate corresponds to the height)	Gliding movement

In the 13 expert raters’ answers we identified 11 distinct movements errors and named them precisely. The 11 error patterns were now assigned to the shot put phase in which this error pattern was most pronounced. An overview of the 11 different error patterns is given in Table 1.

The first purpose of our study was to check if the errors identified by the expert raters could be detected in the MoCap data. For this purpose, we compared the participants and the model and looked for salient deviations in the respective kinematic functions. However, due to different execution durations and velocities of the different shop puts we first had to normalize the kinematic functions to an equal duration. Therefore, four distinct video frames were determined, the beginning of the glide movement, the end of the glide movement, the reaching of the power position and the delivery of the shot (13). We determined these time points using the avatar and the three-dimensional view of the MVN Analyse program. We determined the beginning of the gliding movement by the body's centre of gravity and its course in the direction of movement, i.e., the point in time was unambiguously determined when the centre of gravity moved only in the direction of the shot put. The end of the gliding movement was determined by the planting of the right leg. The power position was reached right before the shot leaves the cheek for the put. With the arm fully extended and the wrist folded down, the moment of push-off was defined.

The further procedure now included the determination of the corresponding biomechanical variables, which are suitable for the analysis of the erroneous pattern. For each of the 11 errors, the appropriate position of body segment or joint angle was selected, for which extension, bending or rotation best visualizes the error image. For some errors, a combination of several body segments and/or joint angles was necessary. Table 1 gives an overview of the error pattern, the variables, and the corresponding phase of interest by which the errors were analysed.

In addition, for the occurrence of each error pattern during the movement, the corresponding phase was defined, which is always framed by two of the four defined time points. They were the gliding movement, reaching the power position, and reaching the push-off position. The corresponing phases was referred to as the phase of interest, the analyzed participant was referred to as “error model”. To compare the kinematic curves of the model with those of the error model, we streched or compessed the curves of the error model, so that the phase of interest had the same extension for the model and the error model and thus could be compared qualitatively. In this way we determined whether the error patterns mentioned by the experts can actually be detected in the kinematic functions.

The two participants whose expert feedback was kept secret will be referred to as Participant 1 and Participant 2 throughout the paper. To answer the second research question, the data of the two participants were examined for the 11 error patterns. This means that there was again a comparison with the model to identify a possible deviation from the technique model. To assess the extent of a possible error, the execution was additionally compared to that of the error model. Objective feedback was created based on these qualitative comparisons.

Before the third research question could be answered, the previously unknown, secret expert feedback of the two participants was evaluated. The comparison of the subjective expert feedback with the objective feedback from Xsens MVN Analyse represented the final methodological step of this investigation.

### Investigation tools

The MVN Link motion suit from Xsens records data on the position, speed and acceleration of body parts and the body's centre of gravity. It also measures data on rotation (internal/external, axial) and extension (flexion/extension) of all joints.

### Data evaluation

The MVN Link motion analysis suit provides a wide variety of data with the associated MVN Analyse program from Xsens. These data can be displayed in the program as a graph. In addition, a three-dimensional avatar is visible in MVN Analyse, which, through calibration, performs the movement of the shot put in exactly the same proportions as the participant. In contrast to the video, MVN Analyse does not only show the movement fixed from one side but can be viewed from any point in space.

The global reference frame and coordinate system are defined so that the *x*-axis is in the direction of motion, the *y*-axis is perpendicular to the direction of motion and oriented to the left, and the z-axis is perpendicular to the direction of motion and oriented upward.^1^ This reference frame applies to the position of the body parts. The calibration process ensured that the right heel establishes the origin (0,0,0) at the beginning of the movement.

To define the joint angles, a different reference frame is used, which is adapted to the anatomical posture of the body. Here, the origin indicates the centre of rotation of the joint. The *x*-axis runs forward, the *y*-axis upward from joint to joint, and the z-axis points to the right. For example, extension of the knee is measured by rotation about the BZ axis of the lower leg with respect to the thigh (B here indicates the joint origin). Abduction/adduction is defined by the rotation around the B*X* axis and internal/external rotation, by that of the BY axis.

During data evaluation, joint angles were analysed in ZXY orientation. Here, flexion/extension of the knee joint by 0 degrees corresponds to full extension of the leg and flexion/extension by 180 degrees would mean full flexion. Since full flexion is not possible, values of up to 160 degrees are realistic here.

For the joint angle to the vertical pelvis, it should be noted that the angle is measured between two axes. One is the axis that is perpendicular to the x-y plane and passes through the pelvis, and the other is the axis that passes through the pelvis and the torso. The angle indicating flexion/extension is positive when the upper body is bent forward. When the upper body becomes supine, the angle changes its orientation and becomes negative.

At this point it should be noted that in Xsens MVN Analyse the position of the ball of the right or left foot cannot be displayed. Since the position of the ball of the foot is of interest in motion analysis, the position of the toes of the right and left feet, respectively, will be used in the course of the data analysis as an approximation for the position of the ball of the foot. Because of their simpler designation, they will be referred to as right and left toes, respectively. Similarly, the position of the right elbow cannot be represented in appropriate diagrams. This is approximated with the position of the right forearm.

## Results

### Qualitative analysis: exemplary illustration of four error patterns

The examination of four out of the eleven error patterns will now be exemplarily illustrated in order to show the feasibility of detecting the experts’ error patterns in the MoCap data. These error patterns will be of relevance for the qualitative analyses concerning Participant 1 and 2, as will be shown later.

#### Error pattern 4: open upper body during the gliding motion

The fourth defect pattern is examined with the help of the T1-C7 joint and C1 head. T1-C7 refers to the connection between two vertebrae of the spinal column. This joint is intended to represent the twisting of the upper body. T1 stands for the first thoracic vertebra and C7 for the seventh (last) cervical vertebra. C1 head denotes the connection of the first cervical vertebra with the head and is thus supposed to represent the untwisting of the head. Here, the axial rotation of the joint connections during the angular motion is of crucial importance. Ideally, the axial rotation and thus the untwisting of the upper body or head is kept as low as possible during the Gliding movement (13).

**Figure 1:**
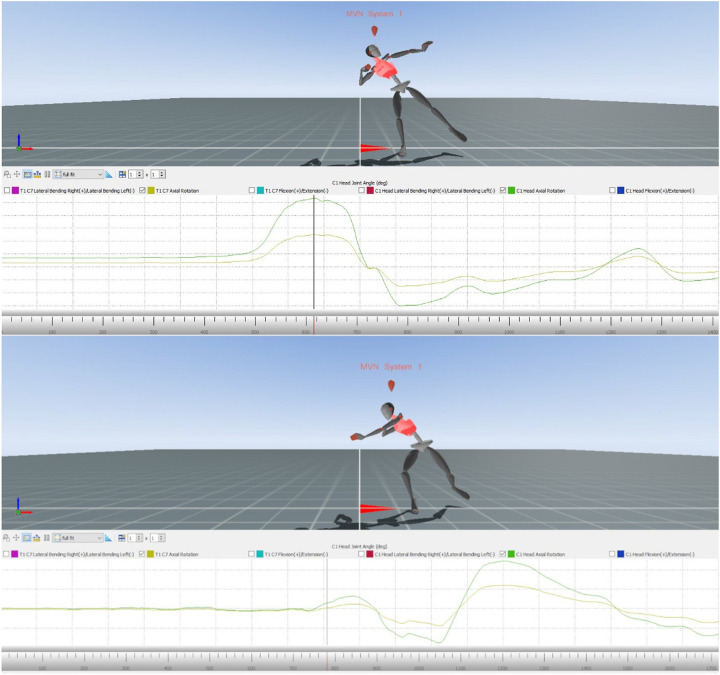
Avatar of the error model (upper part) and the model (lower part) for error pattern 4.

The following images from Xsens MVN Analyse show the avatar of the error model and the model at the end of the glide motion. In the image of the avatar, it is visible that the upper body of the error model is very upturned and the head is already pointing in the direction of motion (Figure 1). In the image of the avatar of the model, the upper body is still closed and the head points in the opposite direction of movement.

**Figure 2:**
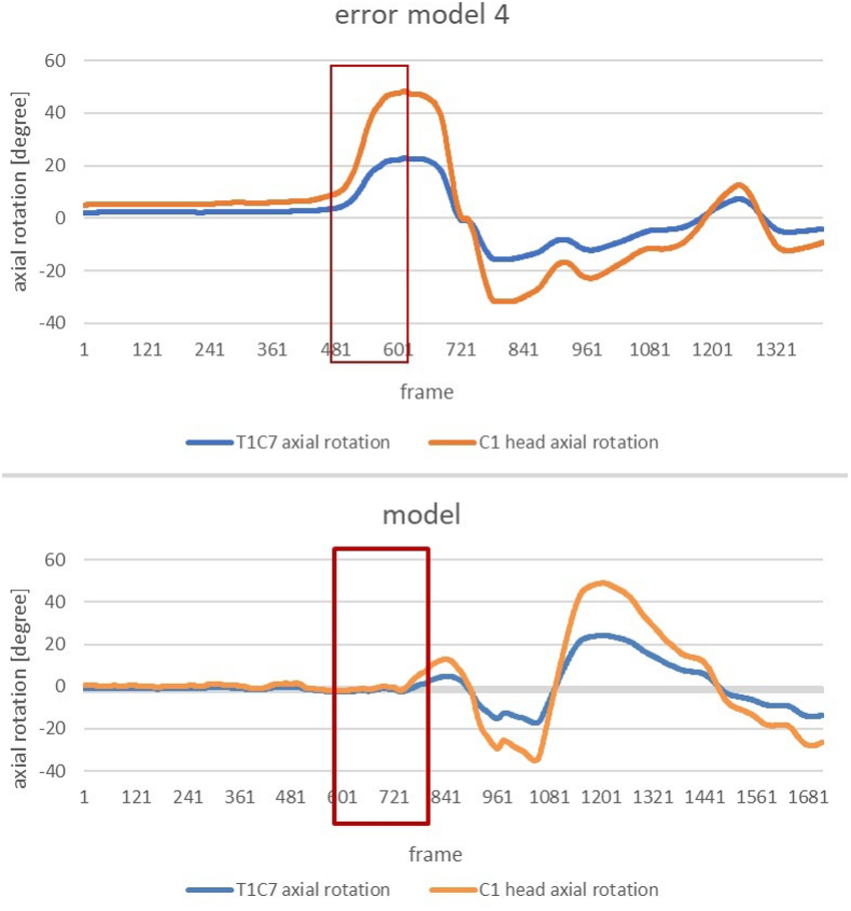
Change in axial rotation of the T1-C7 and C1 head joints of the error model (upper part) and the model (lower part) during the overall movement. Here an in the following figures the x-axis shows frames; the frame rate was 240 frames per second, so 24 frames correspond to 1/10 of a second.

In order to be able to compare the concrete angular dimensions, it is useful to analyse the next diagram, which represents the change in axial rotation of the T1-C7 joint and C1 head during the entire movement of the error model (Figure 2). The area marked in red indicates the phase of interest within the entire course. This starts with the beginning of the slip motion and continues until the end of the slip motion.

**Figure 3:**
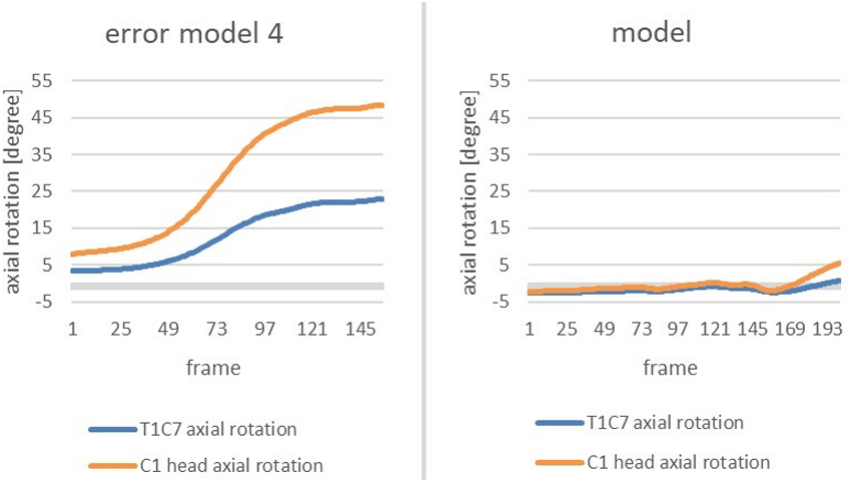
Change in axial rotation of the T1-C7 (blue) and C1 head joint (orange) in the course from the beginning of the movement to the end of the gliding motion of the error model (left) and the model (right) in comparison.

In order to clearly highlight the conspicuous features, the subsequent figure allows a comparison of the change in axial rotation of the two joint connections during the phase of interest of the error model and the model (Figure 3). The extreme deviation between the error model and the model is striking. While the error model rotates open immediately after the start of the slip motion and, at the end of the slip motion, already shows an axial rotation of the C1 head joint of approx. 47 degrees and an axial rotation of the upper body of approx. 24 degrees, the model behaves almost completely closed and only rotates open minimally at the end of the slip motion.

#### Error pattern 8: Too little distance between right and left foot when reaching power position

By determining the position of the toes of the right and left foot as an approximation for the ball of the foot, the eighth error pattern can be detected. Of interest is the comparison of the y-coordinate of the left toe and the right toe, respectively. If no sufficient distance between the right and the left ball of the foot is achieved prior to the power position, an interference for the athlete's rotation of the upper body occurs, and thus the athlete is blocking him/herself [cf. (13)].

**Figure 4:**
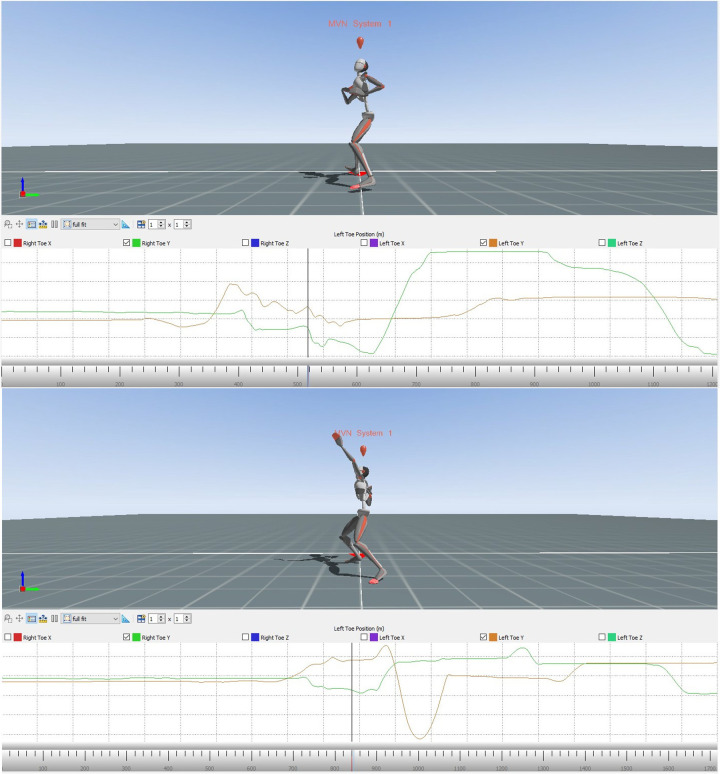
Avatar of the error model (upper part) and the model (lower part) for error pattern 8.

The following images from Xsens MVN Analysis show the error model and the model (Figure 4) when reaching the power position. Comparing the avatar of the error model and the model, it becomes evident that the model's distance between right and left toe is reasonably greater than that of the error model's.

**Figure 5:**
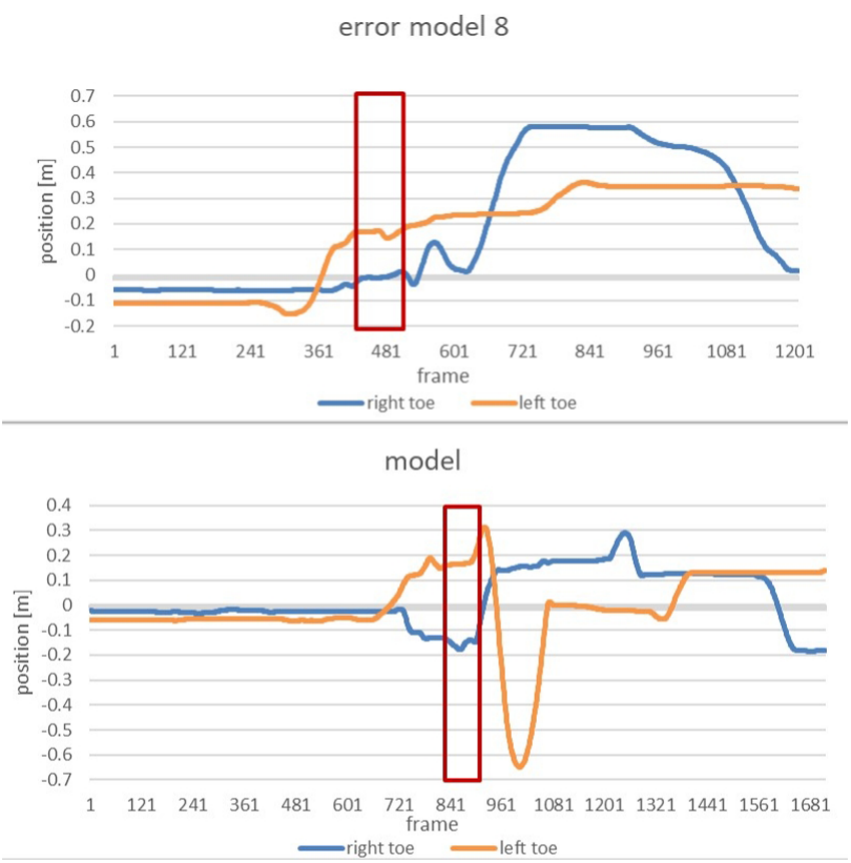
Alteration of the position of the right and left toe of the error model (upper part) and the model (lower part) during the whole movement.

The following two diagrams allow a more precise comparison of the positioning of the right and left toe. The graphs are showing the alterations of the y-coordinates of the right and the left toes during the whole movement for the error model and the model (Figure 5). The area marked in red illustrates the phase of interest during the entire course, starting with the end of the slip motion until reaching the power position.

**Figure 6:**
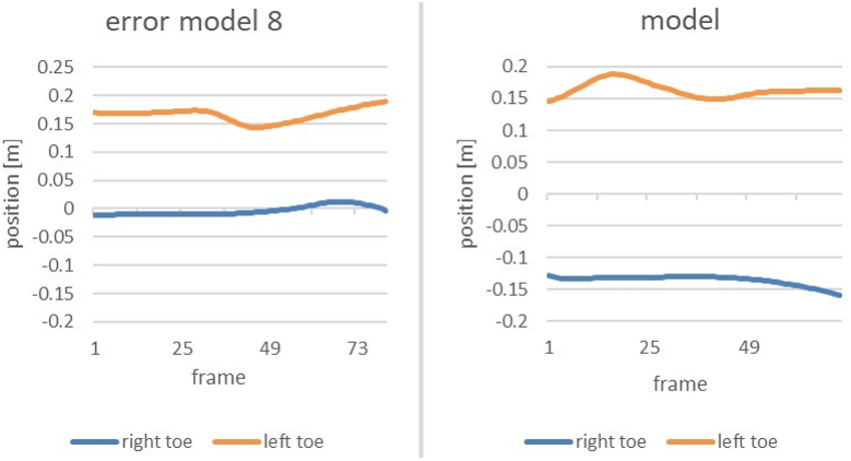
Comparison of the alteration of the position of the right toe and left toe of the error model (left) and the model (right) during the time of the end of the gliding motion up to the power position.

Zoomed in, the subsequent figure show the phase of interest of both the error model and the model (Figure 6).

The vertical axis displays the position of the right and left toe, respectively, whereas the horizontal axis marks the course of the movement from the end of the slip motion up to reaching power position. It is clearly recognizable that the model achieves a distance of 30 cm between right and left food, thus double the distance of that of the error model (15 cm).

#### Error pattern 9: right leg is not turned in when reaching the power position

The ninth error pattern is examined using the joint angle of the right hip. During the shot put movement it is crucial to rotate the right leg slightly inwards when reaching the power position to enable the most powerful possible rotation-extension movement of the legs [cf. (13)]. For this matter, the internal-external rotation of the hip joint during the power position is of decisive importance.

**Figure 7:**
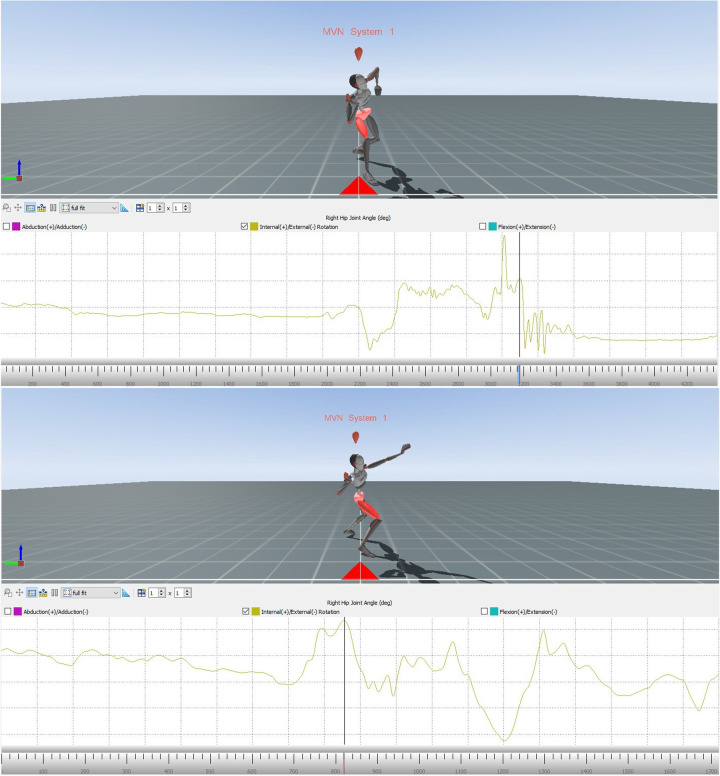
Avatar of the error model (upper part) and the model (lower part) for error pattern 9.

The subsequent images from the Xsens MVN Analysis show the error model and the model when reaching power position (Figure 7). Compared to the model, the error model's right foot points in the opposite direction than the direction of the throw, indicating that its right leg is not turned inwards as it should be.

**Figure 8:**
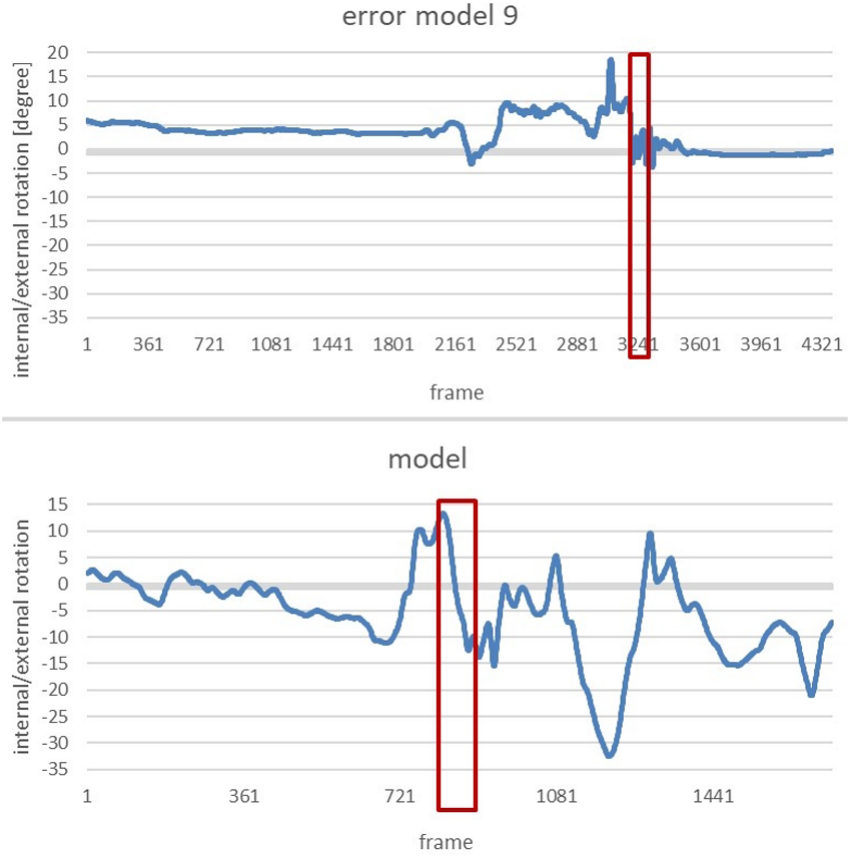
Alteration of the internal/external rotation of the right hip joint of the error model (upper part) and the model (lower part) during the whole movement.

Showing the changes in the external/internal rotation of the right hip joint of the error model and the model (Figure 8) during the movement, these diagrams provide data for comparing the angular dimension of the hip joint. The area marked in red illustrates the phase of interest, that is at the end of the slip motion up to reaching the power position.

**Figure 9:**
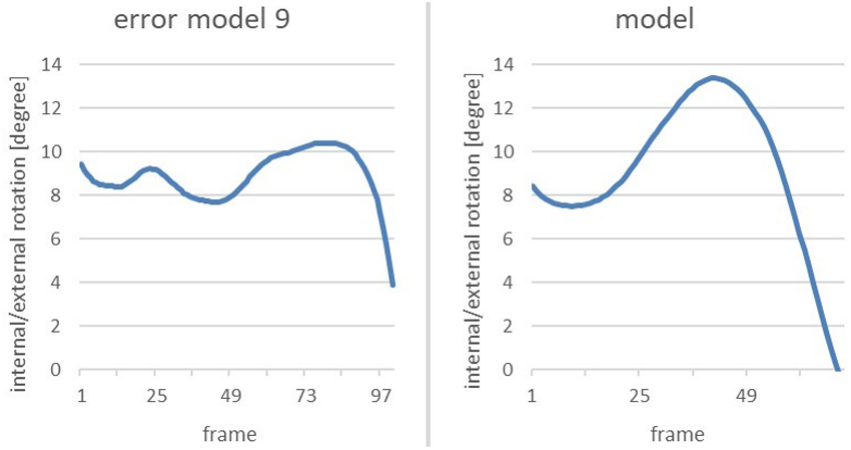
Comparison of the alteration of the internal/external rotation of the right hip joint of the error model (left) and the model (right) from the end of the gliding motion up to power position.

Again, the phase of interest is compared in the next two diagrams (Figure 9). The vertical axis shows the internal/external rotation while the horizontal one illustrates the interesting period during the whole movement.

Both the error model and the model reach a similar internal rotation of the hip joint at the end of the slip motion (9° and 8°, respectively). Yet, when reaching the power position, the model displays a considerably larger internal rotation of 13°, compared to 10° of the error model.

#### Error pattern 10: pelvis is not upright when reaching the power position

This error pattern can be examined by analysing the *vertical pelvis*. The vertical-pelvis is defined as the angle between the axis which runs through the pelvis and the upper body, and the axis which runs perpendicular to the x-y plane and through the pelvis. The flexion or extension of the vertical pelvis during the power position is of crucial importance. Only an upright position of the pelvis (hence, the smallest possible angle of the vertical pelvis) leads to good transfer of power during the shot [cf. (13)].

**Figure 10:**
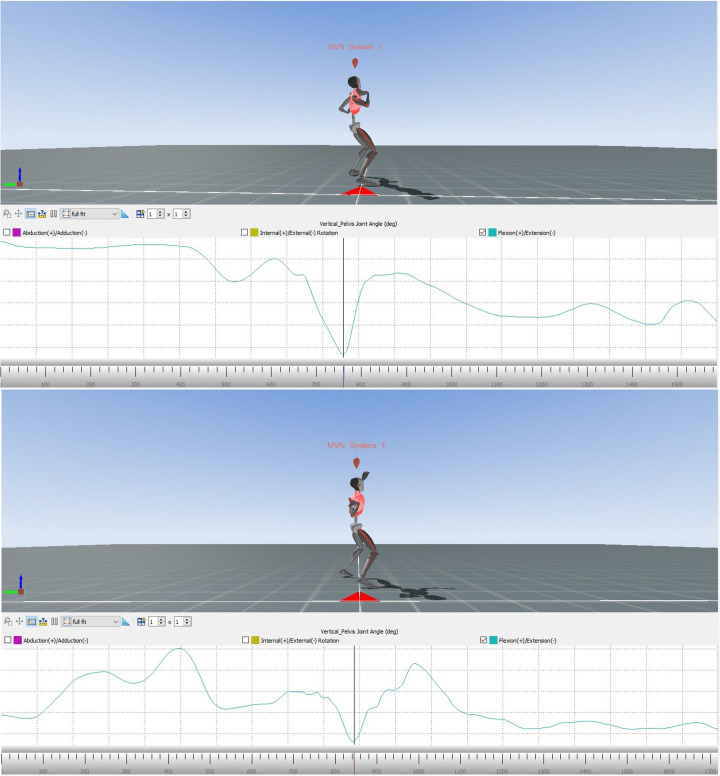
Avatar of the error model (upper part) and the model (lower part) for error pattern 10.

The images from Xsens MVN analysis show the avatars of the model and the error model (Figure 10). It is noticeable that the error model does not reach an upright position during the power position and is rather showing a rounded lower torso. The image of the model shows an assumably upright body, yet this shall be further demonstrated by comparing the angular dimensions.

**Figure 11:**
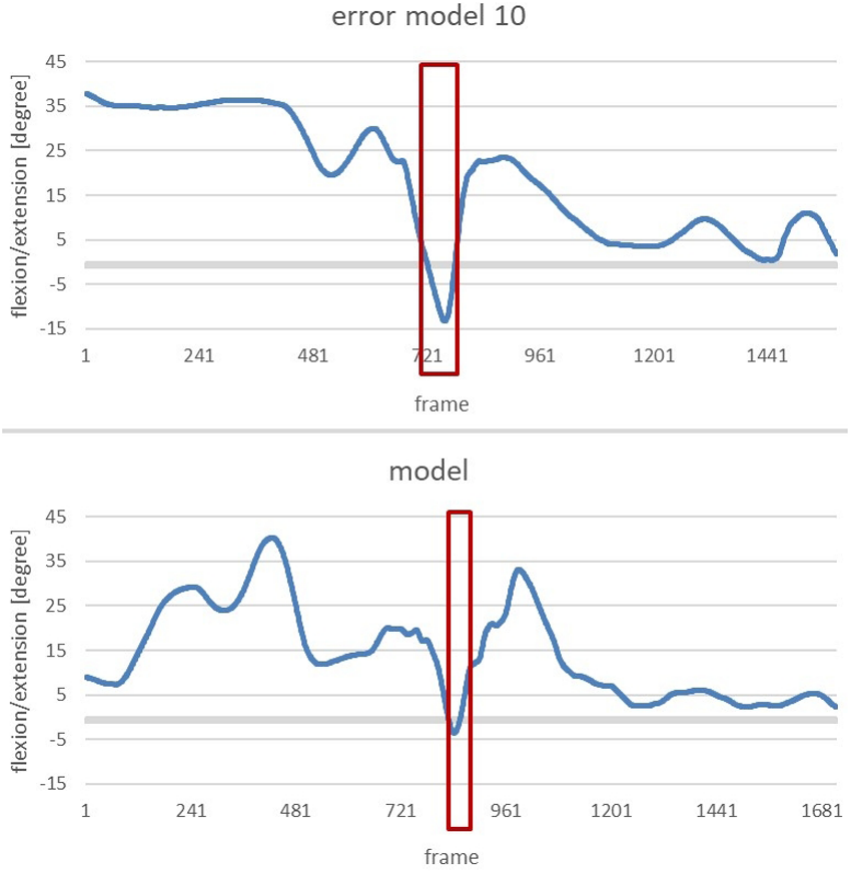
Alteration of the flexion/extension of the vertical pelvis of the error model (upper part) and the model (lower part) during the whole movement.

The subsequent diagrams illustrate the alterations of extension/flexion of the vertical pelvis of the error model and the model (Figure 11). The phase of interest (marked in red) starts with the end of the gliding motion up to the power position.

**Figure 12:**
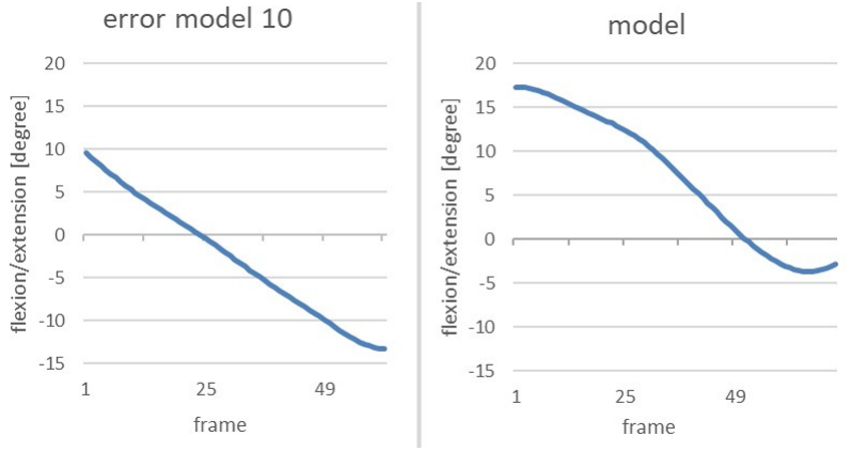
Comparison of the alteration of the flexion/extension of the vertical pelvis of the error model (right) and the model (left) from the end of the gliding motion up to power position.

To clearly highlight the anomalies, the next figure shows a comparison of the change in flexion/extension of the vertical pelvis of the error model and the model during the phase of interest (Figure 12).

The error model shows a flexion of 10 degrees at the end of the gliding phase; thus, the pelvis is flexed forward. In the subsequent course of the movement, the upper body extends beyond the vertical axis of the pelvis which renders the angle negative through the other fixation point. This can be interpreted as the rounded lower torso which was already visible when analysing the image of the avatar. The curve of the model can also be interpreted as an uprising of the upper body, yet no supine position is reached. A total upright position would be achieved by reaching 0 degrees, so the −4 degrees of the model can be seen as an almost upright position of the pelvis when reaching the power position.

### Qualitative analysis: participant 1 and participant 2

In the previously described manner, all the eleven error characteristics outlined by the expert raters could be detected in the MoCap Data. In a second step, the MoCap Data of Participant 1 and Participant 2 was now used to determine whether by analysis of the data, the main and second error pattern could be identified. These error patterns correspond to the previously described ones. To further prove the validity of these results, i.e., whether indeed the objectively best KP was found, the results were then compared to the feedback given by the expert raters. In case of consensus between the results obtained by analysing the data and the expert raters’ feedback, the results of the Xsens MVN Analysis will be interpreted as valid.

#### Results for participant 1

To determine whether an error pattern was present, the data of Participant 1 was examined for possible deviations by comparing it with the technique model in each of the relevant areas (the explicit method was shown in the previous with the error model and the model). In this way, six of the 11 error patterns could be identified in the movement of Participant 1. To further assess the extent of a possible error, Participant 1 was also compared to the error model. As the main error, the *error pattern 9: Right leg is not turned in when reaching power position* was identified.

A more detailed analysis of the data revealed that this error patterns is even more severe with Participant 1 than it is with the error model. Whereas the figures of the model and the error model are positive, which means that the hip was rotated inwards during the end of the gliding movement, the figures of Participant 1 are negative. This gives reason to assume that the person was not turning the hips inwards but rather outwards at the end of the slip motion. The image of the avatar confirms this assumption.

**Figure 13:**
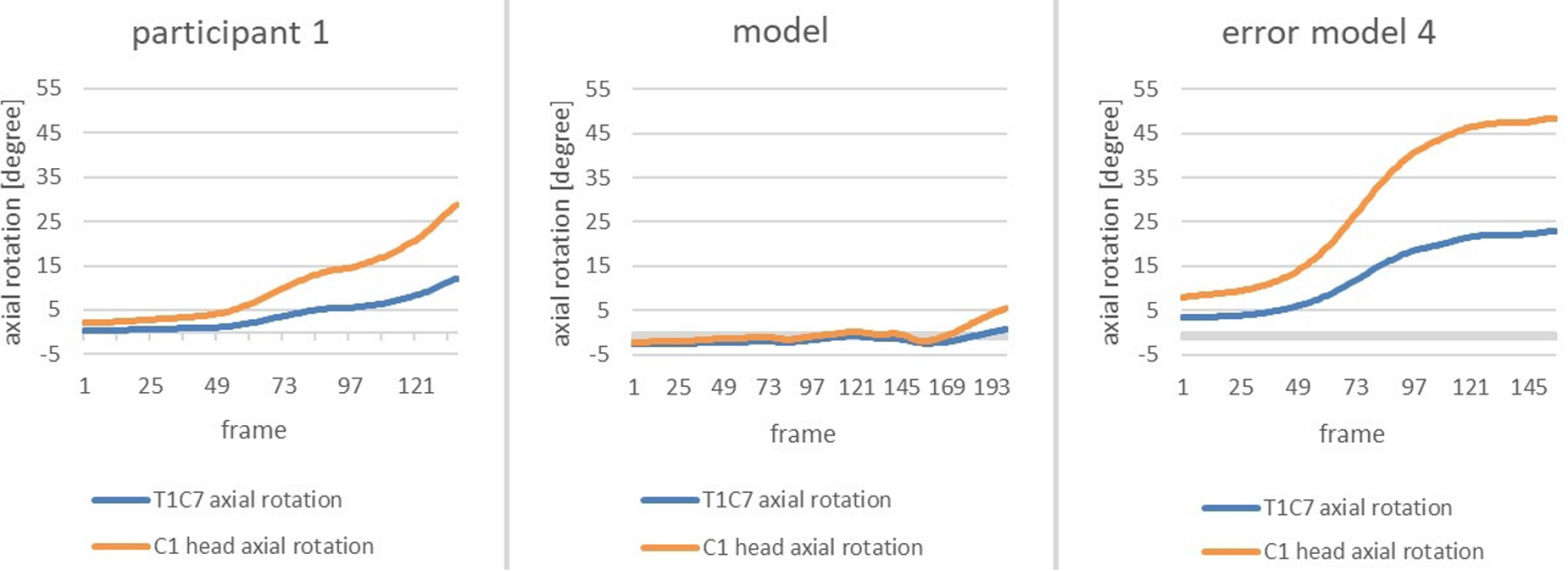
Change in axial rotation of the T1-C7 and C1 head joints of the participant 1 (left), the model (middle) and the error model (right) from the beginning to the end of the gliding motion.

As the second main error, *error pattern 4: open upper body during the gliding motion* was found. Again, the figure shows the data of the error model, the model and Participant 1 (Figure 13).

By comparison with the model, it becomes evident that the error pattern is present as the axial rotation of the head is more prominent. However, compared to the error model, the figures are smaller than that of the error model.

#### Results for participant 2

**Figure 14:**
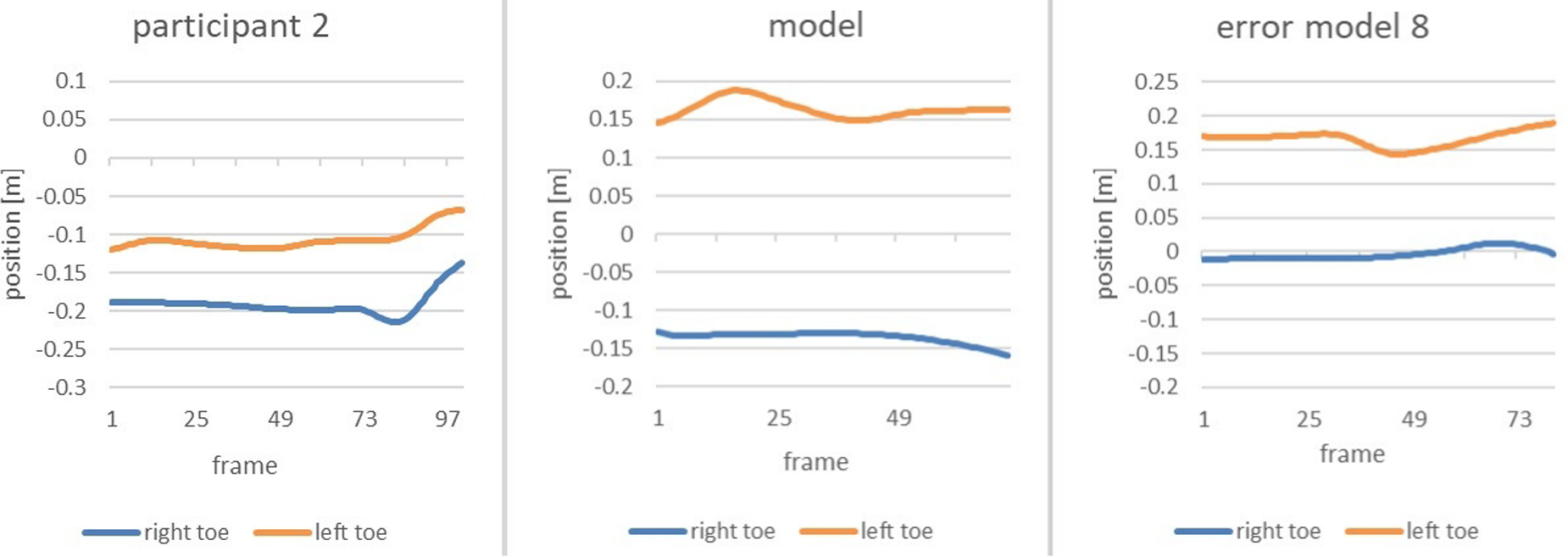
Comparison of the alteration of the position of the right toe and left toe of participant 2 (left), the model (middle) and the error model (right) during the end of the gliding motion up to the power position.

The same efforts as for Participant 1 were undertaken for Participant 2. For two out of the eleven error patterns, the movement of Participant 2 deviated strongly from that of the technique model. As the main error the *error pattern 8: Too little distance between right and left foot when reaching power position* was detected. The following figure shows a comparison of Participant 2, the error model and the model (Figure 14).

The distance between right and left foot is significantly smaller than that of the model. With only 10 cm it is even smaller than that of the error model, rendering the error in the movement of Participant 2 noticeably severe.

**Figure 15:**
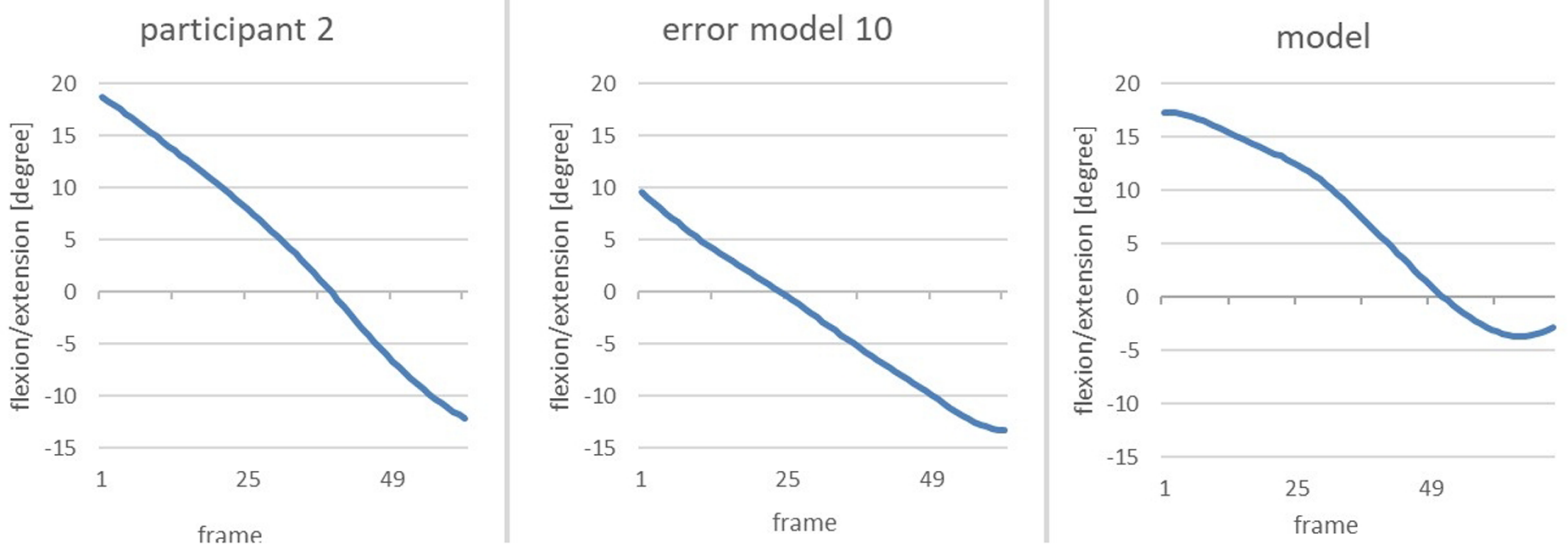
Comparison of the change in flexion/extension of the vertical pelvis from the end of the gliding movement until the power position of participant 2 (left), the model (middle) and the error model (right).

As the second main error, *error pattern 10: pelvis is not upright when reaching power position* was identified. The following image shows the flexion/extension of the vertical pelvis of the Participant 2, the error model and the model (Figure 15).

When reaching power position, the figures of Participant 2 are negative, meaning that the pelvis is tilted backwards at a larger rate than that of the model (−12 degrees compared to −4 degrees). Compared to the error model, it becomes obvious that error pattern 10 is even more profound in Participant 2 than in the error model.

### Prove of validity for the results: comparison to expert raters’ feedback

So far, we could demonstrate that it was indeed possible to examine the MoCap data of Participant 1 and Participant 2 to determine the presence as well as the severity of error patterns. As the last methodological step, we wanted to assess the validity of the objective feedback generated through Xsens MVN Analysis by comparing it to the expert raters’ subjective feedback. The expert raters’ feedback to Participant 1 and Participant 2 was concealed up to this point and was evaluated only after the analysis of the Participants’ MoCap Data.

The subsequent table (Table 2) displays the error patterns detected through the Xsens MVN Analysis (objective feedback) as well as the ones identified by expert rater 1 and 2 (subjective feedback).

**Table 2:** Objective and subjective feedback for participant 1 (the expert raters’ feedback is translated literally from German into English).

	Xsens MVN analysis	Expert 1	Expert 2
Main error	Right leg is not turned in when reaching power position	Position of the right foot points too much backwards	Stretching movement of the left leg not pronounced enough
Second main error	open upper body during the gliding motion	Looking too early in the direction of impact	Right leg is not turned in

Even though the formulation of the error patterns differs, the objective feedback “Right leg is not turned in when reaching power position” matches with the experts’ feedback of “Position of the right foot points too much backwards” and “Right leg is not turned in”. Similarly, “Looking too early in the direction of impact” corresponds to “Open upper body during the gliding motion”. It can be concluded that it was possible to detect the error patterns named by the experts through the Xsens MVN Analysis. However, the error pattern “Stretching movement of the left leg is not pronounced enough” could not be confirmed through the analysis.

For Participant 2, we again compared the two error patterns found by Xsens MVN Analysis with the error patterns suggested by the expert raters (Table 3).

**Table 3:** Objective and subjective feedback for participant 2 (the expert raters’ feedback is translated literally from German into English).

	Xsens MVN analysis	Expert 1	Expert 2
Main error	Too little distance between right and left foot when reaching power position	No upright hip position	Ball is away from the neck
Second main error	pelvis is not upright when reaching power position	Rotation of the right leg and the right shoulder around the left side of the Body is not pronounced enough	Bend in the upper body

Once more, it is mainly the formulation of the error patterns that differs. The error patterns “no upright hip position” and “bend in the upper body” by the experts do correspond with “pelvis is not upright when reaching power position” identified as the main error by Xsens MVN Analysis.

The error patterns “Ball is away from neck” and “Rotation of the right leg and the right shoulder around the left side of the Body is not pronounced enough”, however, could not be identified through Xsens MVN Analysis. Nonetheless, there were anomalies in the data considering error pattern 5 and error pattern 9 that can be interpreted as the respective error pattern identified by the expert raters.

To conclude, it can be stated that the objective feedback generated through Xsens MVN Analysis is to a good extent in accordance with the expert raters’ subjective feedback. We were able to demonstrate that through this programme, the main and second error of the participants could be identified. Thus, with some limitations, the objectively best KP for the participants could be applied.

## Discussion

In this article we argue that the validity of experimental studies on feedback and instructions can be standardized using MoCap data. As external KP-feedback is often dependent on subjective expertise, programmes such as Xsens MVN Analysis provide an alternative approach to generate objective feedback. In this section, we provide further discussion concerning the limitations and possibilities of generating feedback through MoCap data.

### Limitations

Due to the limited data available for the analysis of the error patterns, only the eleven error patterns identified by the expert raters could also be detected in the data. We do not assume that this covers the entirety of all possible errors. It is possible that persons to be assessed may show other error patterns that cannot be found. Some more data would have to be collected to secure that most possible error patterns occur at least once. As for many movements there are various technique errors, this involves a great effort. Additionally, in our feasibility study, the error patterns were based on the expertise of our raters.

To make the kinematic curves comparable, we have compressed or stretched them within the different phases of the movement. The absolute time required for each phase is lost in the process. However, absolute time can influence the optimum curve characteristics. For further research, in particular error detection by an AI, we want to feed information about the absolute time into the artificial neural network.

In our study, we considered all error patterns to be equally detrimental to achieving the movement goal. The errors were then prioritized based on the size of the deviation from the model. However, this is not necessarily the case.

These limitations can be overcome by analysing a larger number of error models. In addition, the errors would have to be weighted so that a relationship between the size of the deviation from the model and the effect on the achievement of the movement target is considered when prioritizing the errors and providing feedback.

### Outlooks

Once these limitations are overcome, experimental studies on feedback and instructions are no longer tied to the expertise of people and their quality of feedback. An optimised MoCap data analysis has the potential of generating a standardized and reasonable individual KP feedback. As this data would be objective and valid, this in turn would increase the validity of experimental studies.

Furthermore, movement errors that are hidden from the human eye due to velocity of movement or unfavourable perspective can be detected in the data. Otherwise, unnoticed by an expert, MoCap data can provide the external feedback which is needed by an individual. Similarly, data can be gathered which goes beyond human expertise like determining the velocity of sports equipment such as the shot. Velocity of movement and/or sports equipment is often the performance-determining factor.

Finally, our study is a proof of concept that automatized feedback is possible. Given the modern techniques of motion capturing from a 2D video by the assistance of artificial intelligence, our study proves that it is possible to have videos recorded with a cell phone analysed by a program and to give the athlete valuable KP feedback.

In summary, we illustrated that there is still a lack of experimental studies on feedback and instructions using objective and valid KP. The validity of these studies depends on the quality of the KP feedback. However, this gap can be filled by using MoCap data, as we could demonstrate with our feasibility study.

## Conclusion

Several studies on feedback and instructions are referring to the outcome of a movement, i.e., the knowledge of result. In practice, however, external feedback on knowledge of performance plays a larger role than KR. So far, experiments relying on external KP are bound to the expertise of the people providing the feedback. Thus, the quality of the results might be decreased if not the best or even harmful external feedback was given. Previous studies on KP proclaiming standard tasks often lacked real-time feedback (10, 11). Yet, with technology avalanching in the field of automated motion analysis, new ways have opened for researchers to study feedback and instructions.

In our pilot investigation we illustrated that it is indeed possible to find the objectively best—or at least a standardized and thus comparable KP—by using MoCap data, even if some limitations still exist. We showed that all the error characteristics identified by expert raters can also be detected in the data. Furthermore, we were also able to estimate the severity of such error patterns by a comparative analysis with our model. Finally, we could prove the quality and validity of the Xsens MVN Analysis by comparing it to the subjective feedback of the expert raters.

We suggest that if said limitations are lifted, the use of MoCap data could bring experimental studies on feedback and instructions to a more objective level. Even complex movements such as the shot put can be examined in real time. Further research is needed to assess and overcome the limitations regarding the use of MoCap data. Such research will provide a theoretical basis for coming experimental studies on feedback and instructions.

## Data Availability

The raw data supporting the conclusions of this article will be made available by the authors, without undue reservation.

## Footnotes

^1^Movella Technologies B.V. (2024). Xsens MVN User Manual. Available online at: https://www.movella.com/hubfs/MVN_User_Manual.pdf
